# White matter microstructure in a genetically defined group at increased risk of autism symptoms, and a comparison with idiopathic autism: an exploratory study

**DOI:** 10.1007/s11682-015-9496-z

**Published:** 2015-12-23

**Authors:** Marcia N. Goddard, Sophie van Rijn, Serge A. R. B. Rombouts, Hanna Swaab

**Affiliations:** 1 Faculty of Social and Behavioural Sciences, Department of Clinical Child and Adolescent Studies, Leiden University, Wassenaarseweg 52, 2333 AK, Leiden, The Netherlands; 2 Institute of Psychology, Leiden University, Leiden, The Netherlands; 3 Leiden Institute for Brain and Cognition, Leiden, The Netherlands; 4 Department of Radiology, Leiden University Medical Center, Leiden, The Netherlands

**Keywords:** Autism, Klinefelter syndrome, Diffusion tensor imaging, White matter, Structural connectivity, Genetics

## Abstract

Klinefelter syndrome (47,XXY) is associated with physical, behavioral, and cognitive consequences. Deviations in brain structure and function have been reported, but structural characteristics of white matter have barely been assessed. This exploratory diffusion tensor imaging study assessed white matter microstructure in boys with 47,XXY compared with non-clinical, male controls. Additionally, both similarities and differences between 47,XXY and autism spectrum disorders (ASD) have been reported in cognition, behavior and neural architecture. To further investigate these brain-behavior pathways, white matter microstructure in boys with 47,XXY was compared to that of boys with ASD. Fractional anisotropy (FA), radial diffusivity (Dr), axial diffusivity (Da), and mean diffusivity (MD) were assessed in 47,XXY (*n* = 9), ASD (*n* = 18), and controls (*n* = 14), using tract-based spatial statistics. Compared with controls, boys with 47,XXY have reduced FA, coupled with reduced Da, in the corpus callosum. Boys with 47,XXY also have reduced Dr. in the left anterior corona radiata and sagittal striatum compared with controls. Compared with boys with ASD, boys with 47,XXY show reduced Da in the right inferior fronto-occipital fasciculus. Although this study is preliminary considering the small sample size, reduced white matter integrity in the corpus callosum may be a contributing factor in the cognitive and behavioral problems associated with 47,XXY. In addition, the differences in white matter microstructure between 47,XXY and ASD may be important for our understanding of the mechanisms that are fundamental to behavioral outcome in social dysfunction, and may be targeted through intervention.

## Introduction

Boys with 47,XXY (often referred to as Klinefelter syndrome) are born with an extra X chromosome. It affects approximately 1 in 650 newborns (Bojesen et al. 2003) and although it is not associated with marked facial or physical abnormalities, a range of physical, behavioral, and cognitive consequences may be present, to varying degrees (Giltay and Maiburg 2010; Groth et al. 2013). Physical consequences include tall stature, fertility problems, and endocrinological imbalances (Lanfranco et al. 2004; Ross et al. 2005). Behaviorally, an increased risk of psychopathology is often reported. To illustrate, elevated incidences of bipolar disorder, attention-deficit/hyperactivity disorder, and autism spectrum disorders are found among individuals with 47,XXY (Bishop et al. 2011; Bruining et al. 2009; Cederlof et al. 2014; Tartaglia et al. 2010; van Rijn and Swaab 2011). Cognitive problems associated with 47,XXY are heterogeneous and range from subtle to quite pronounced. Intellectual functioning at the lower end of the normal range, language impairment, and executive dysfunction are among the most often reported cognitive characteristics of 47,XXY (Boada et al. 2009; Geschwind et al. 2000; Leggett et al. 2010; Verri et al. 2010). However, it has remained largely unclear what the neural mechanisms are that underlie cognitive impairment and the increased risk of behavioral problems in 47,XXY.

From a neuroscientific perspective, deviations in structure and function of the brain are central to the understanding of both psychopathology and cognitive impairment. It is likely that the genetic effects of 47,XXY on cognition and behavior are mediated by the structure and function of the brain, underlining the importance of studying the neural mechanisms associated with this condition. In recent years, advances in magnetic resonance imaging (MRI) analysis methodology have led to increased knowledge of brain structure and function in 47,XXY (Mueller 2013; Reiss et al. 2000). This condition is associated with deviations in gray matter volume, and functionality of language and social-cognitive brain regions appears to be abnormal (Brandenburg-Goddard et al. 2014; Bryant et al. 2011; Skakkebaek et al. 2013; Steinman et al. 2009; van Rijn et al. 2008; Van Rijn et al. 2012). These types of studies provide better understanding of the neural mechanisms associated with the cognitive and behavioral consequences associated with 47,XXY, contributing to the aim of understanding gene-brain-behavior pathways.

While knowledge regarding deviations in gray matter volume associated with 47,XXY has become increasingly extensive, there is a growing awareness among neuroscientists of the importance of insight into connections between brain areas. Studying neural connectivity is crucial to understanding how information is processed in the brain, and thus to delineating the neural mechanisms associated with the cognitive and behavioral consequences of 47,XXY. One way of looking at brain connectivity, is studying the integrity of neural fiber tracts connecting neurons in different parts of the brain. This provides insight into how adequately neural signals are transmitted and thus how effectively various parts of the brain communicate. Diffusion tensor imaging (DTI) offers the possibility of measuring these tracts in vivo. As nearly fifty percent of the human brain is composed of white matter, which contributes substantially to both cognition and behavior (Filley 2005), DTI may provide unique information regarding structural connectivity in 47,XXY. The only DTI study in 47,XXY thus far focused on adult males, and reported reduced FA in the left posterior limb of the internal capsule, the bilateral anterior cingulate, and the left arcuate bundle (DeLisi et al. 2005). Studying children with 47,XXY may aid in determining if deviations in brain connectivity develop early in life. Therefore, the primary aim of the current study was to exploratively assess neural fiber tract integrity in boys with 47,XXY compared with non-clinical controls, using tract-based spatial statistics (TBSS).

Secondarily, it may be important to assess how deviations in white matter integrity in 47,XXY compare to other (neurodevelopmental) conditions. There is now substantial empirical evidence showing parallels between behavioral symptoms associated with 47,XXY, and those associated with autism spectrum disorders (ASD) (Cederlof et al. 2014; Cordeiro et al. 2012; Tartaglia et al. 2010). However, the specific manifestation of social problems, as well as the underlying cognitive and neural mechanisms associated with social dysfunction, may be different (Brandenburg-Goddard et al. 2014; Bruining et al. 2010; Van Rijn et al. 2014a; Van Rijn et al., 2014b). The discrepancy between behavioral similarities and differences in underlying mechanisms between individuals with 47,XXY and individuals with ASD, highlights the need for more knowledge regarding overlap and differences in brain-behavior pathways in these conditions. In pursuit of this knowledge, the secondary aim of the current study was to compare neural fiber tract integrity in boys with 47,XXY, to that of boys with ASD. As this was the first study to assess white matter integrity in children with 47,XXY four measures (i.e. fractional anisotropy, radial diffusivity, axial diffusivity and mean diffusivity) are reported, to provide a comprehensive overview of neural fiber tract characteristics associated with this condition.

## Method and materials

### Participants

DTI analyses were performed on a subsample of participants from the study by Brandenburg-Goddard et al. (2014). Nine boys with 47,XXY [*M*_age_ = 14.53, *SD* = 3.03, *range* = 11.1–18.9)], eighteen boys with ASD [*M*_age_ = 11.84, *SD* = 2.13, *range* = 9.0–18.2)], and fourteen non-clinical, male controls [*M*_age_ = 11.95, *SD* = 2.91, *range* = 9.2–18.5)] were included in analyses. Analysis of variance (ANOVA) revealed a significant effect of group on age [*F*(2,38) = 3.575, *p* = 0.038], for which post-hoc testing showed this was due to a significant age difference between the 47,XXY group and both the control group (*p* = 0.027), and the ASD group (*p* = 0.016). To control for age related differences in brain maturation, age was used as a confound regressor in DTI analyses. For one boy with 47,XXY, IQ data were missing. A significant main effect of group on overall IQ was found [*F*(2,39) = 7.181, *p* = 0.002], due to a significant difference in IQ scores between the 47,XXY group [*M*_*IQ*_ = 77, *SD* = 10.69, *range* = 57–88] and both the control [*M*_*IQ*_ = 103, *SD* = 15.45, *range* = 74–132] and ASD groups [*M*_*IQ*_ = 97, *SD* = 18.15, *range* = 68–123], with the 47,XXY group having lower mean IQs than controls (*p* = 0.002) and the ASD group (*p* = 0.014). Descriptive statistics for age and IQ are summarized in Table 1. Within the 47,XXY group, four participants received supplemental testosterone treatment at the time of the study. Five participants did not receive supplemental testosterone treatment.

**Table 1:** Mean age and IQ (including standard deviation and range) of the 47,XXY, ASD and control groups

Group	Age			IQ		
	Mean	SD	Range	Mean	SD	Range
47,XXY	14.53	3.03	11.1–18.9	77	10.69	57–88
ASD	11.84	2.13	9.0–18.2	97	18.15	68–123
Controls	11.95	2.91	9.2–18.5	103	15.45	74–132

The 47,XXY group was recruited using various strategies, to avoid recruitment bias as much as possible. The sample consisted of children who were actively followed up after prenatal diagnosis with the help of clinical genetics departments in The Netherlands and Belgium, as well as children whose parents actively sought information about the condition of their child (recruited through support groups and calls for participants, with the help of the Dutch Klinefelter Association), and those who were seeking help for developmental problems (recruited through pediatricians, psychologists, psychiatrists and clinical genetics departments). The ASD group was recruited through the Center for Autism, a pediatric psychiatric outpatient department in The Netherlands. All boys with ASD were classified according to the DSM-IV criteria (A.P.A. 1994) using the Autism Diagnostic Interview-Revised (ADI-R) (Lord et al. 1994) parental questionnaires, parental interviews, developmental history and family history, information from primary physicians as well as elaborate expert clinical observations. All ASD diagnoses were reached through consensus among a multidisciplinary team of mental health professionals, including board-certified pediatric psychiatrists with experience in the field of autism. As part of routine clinical care, all participants in the ASD group underwent somatic and psychiatric screening performed by medical professionals. In none of the children with ASD, genetic karyotyping was warranted as a result of this screening. Because genetic diagnostic screening without clinical indication was considered unethical, no further genetic testing was done.

Non-clinical controls were recruited through schools in the western part of The Netherlands and screened for psychopathology. None scored in the clinical range (>70) on the Child Behaviour Checklist (CBCL) (Achenbach 1991).

Inclusion criteria for all participants were Dutch as primary language and age between ten and eighteen years. Exclusion criteria were a recent history of substance abuse, intellectual disability (<60 IQ points), scan or motion artifacts, as well as neurological conditions (e.g. structural brain damage due to prenatal/birth complications, tumors, strokes or diseases affecting the central nervous system). All participants and their parents received a complete description of the study and provided written informed consent prior to participation, in accordance with the Declaration of Helsinki. All children received a gift card for participation, and travel costs were reimbursed. The experiment was approved by the Ethical Committee of the Leiden University Medical Center, Leiden, The Netherlands.

### Procedure

All scans were administered in one morning or afternoon at the Leiden University Medical Center (Leiden, The Netherlands). Upon arrival, participants were screened for metals or other dangerous physical conditions using the MRI safety check list. Subsequently, they were escorted to the mock scanner, which was used to acclimate participants to the scanner environment. Participants were allowed to spend as much time as needed in the mock scanner.

### MRI data acquisition

Scanning was performed on a 3-Tesla Philips Achieva whole body MRI scanner (Philips Healthcare, Best, The Netherlands), using an 8-channel SENSE receiver head coil. All anatomical scans were reviewed and cleared by a radiologist. No anomalous findings were reported. DTI scans were acquired as part of an MRI sequence including anatomical and functional scans, using a single-shot echo-planar imaging (EPI) sequence with the following parameters: TR = shortest, TE = 56 ms, flip angle 90°, b factor = 1000 s/mm^2^, voxel dimensions =2.3 mm isotropic, 73 slices, no slice gap. DTI scans were acquired along sixteen directions, together with a baseline imaging having no diffusion weighting (b = 0). The total DTI acquisition time was approximately six minutes.

### Outcome measures

#### Autism spectrum symptoms

The Social Responsiveness Scale (SRS) (Constantino and Gruber 2005) is a 65-item parent-report questionnaire that was used to assess the degree of autism spectrum symptoms. It includes items that ascertain social awareness, social cognition, social communication, social motivation, and autistic mannerisms. Higher scores indicate stronger autism traits. A validation study (Constantino et al. 2003) indicated that the SRS was highly correlated with the Autism Diagnostic Interview-Revised (ADI-R) (Lord et al. 1994). Coefficients were higher than 0.64 between SRS scores and all ADI-R scores. Total SRS scores were used as an indication of autism spectrum symptoms.

#### DTI analysis

##### Preprocessing

The DTI data of all participants were preprocessed using FSL (FMRIB’s Software Library, http://fsl.fmrib.ox.ac.uk/fsl/fslwiki) version 5.0.4 (Jenkinson et al. 2012; Smith et al. 2004a). Affine registration of each diffusion weighted image to the b = 0 reference image was performed to correct for distortion and motion artifacts induced by eddy currents or head motions, followed by non-brain tissue removal (Smith 2002). To generate individual FA, Dr., Da, and MD maps for each participant, the diffusion tensor model was fitted to each voxel using FMRIB’s Diffusion Toolbox (Behrens et al. 2003). For Da, the principal eigenvalue (L1) was used, for Dr. the two minor eigenvalues (L2 and L3) were averaged. MD was calculated as the average of the three eigenvalues (L1, L2, and L3).

##### Tbss

Fractional anisotropy (FA), an expression of the directionality of white matter tracts, radial diffusivity (Dr), an indication of myelination, axial diffusivity (Da), an indication of axonal integrity, and mean diffusivity (MD), the average diffusion of water within white matter tracts, together provide a quantification of white matter integrity. Voxelwise statistical analysis of the FA data was carried out using TBSS (Smith et al. 2006), part of FSL (Smith et al. 2004b). First, FA images were created by fitting a tensor model to the raw diffusion data, and then brain-extracted (Smith 2002). All participants’ FA data were then aligned into a common space using the nonlinear registration tool (Andersson et al. 2007), which uses a b-spline representation of the registration warp field. Next, the mean FA image was created and thinned to create a mean FA skeleton which represents the centres of all tracts common to the group. The mean FA skeleton was thresholded at an FA value of ≥0.35 to exclude peripheral tracts and minimize partial voluming. Each participant’s aligned FA data was then projected onto this skeleton. Similarly, Dr., Da, and MD data were projected onto the skeleton using FA registration and skeleton projection parameters. The resulting FA, Dr., Da, and MD data were fed into voxelwise permutation-based analysis, using Randomise (Nichols and Holmes 2002; Winkler et al. 2014), with a general linear model including four contrasts (47,XXY < CON and 47,XXY > CON for the first aim; 47,XXY < ASD and 47,XXY > ASD for the second aim), age as a confound regressor, and 5000 permutations, correcting for multiple comparisons across space using threshold-free cluster enhancement (TFCE, *p* < 0.05) (Smith and Nichols 2009).

## Results

### Autism spectrum symptoms

A significant effect of group on SRS scores was found [*F*(2,31) = 23.61, *p* < 0.001], with mean scores in both the 47,XXY [*N* = 6; *M*_srs_ = 74.2 (*SD* = 32.5)] (*p* = 0.005) and ASD [*N* = 14; *M*_srs_ = 98.3 (*SD* = 34.1)] (*p* < 0.001) groups being significantly higher than in controls [*N* = 14; *M*_srs_ = 27.6 (*SD* = 15.0)]. No significant difference in mean scores between the 47,XXY and ASD groups was found. In the 47,XXY group, T-scores suggested two participants scored in the normal range, one scored in the mild to moderate range, while three scored in the severe range. In the ASD group, T-scores suggested one participant scored in the normal range, three scored in the mild to moderate range, while ten scored in the severe range.

### TBSS: 47,XXY versus controls

**Fig. 1:**
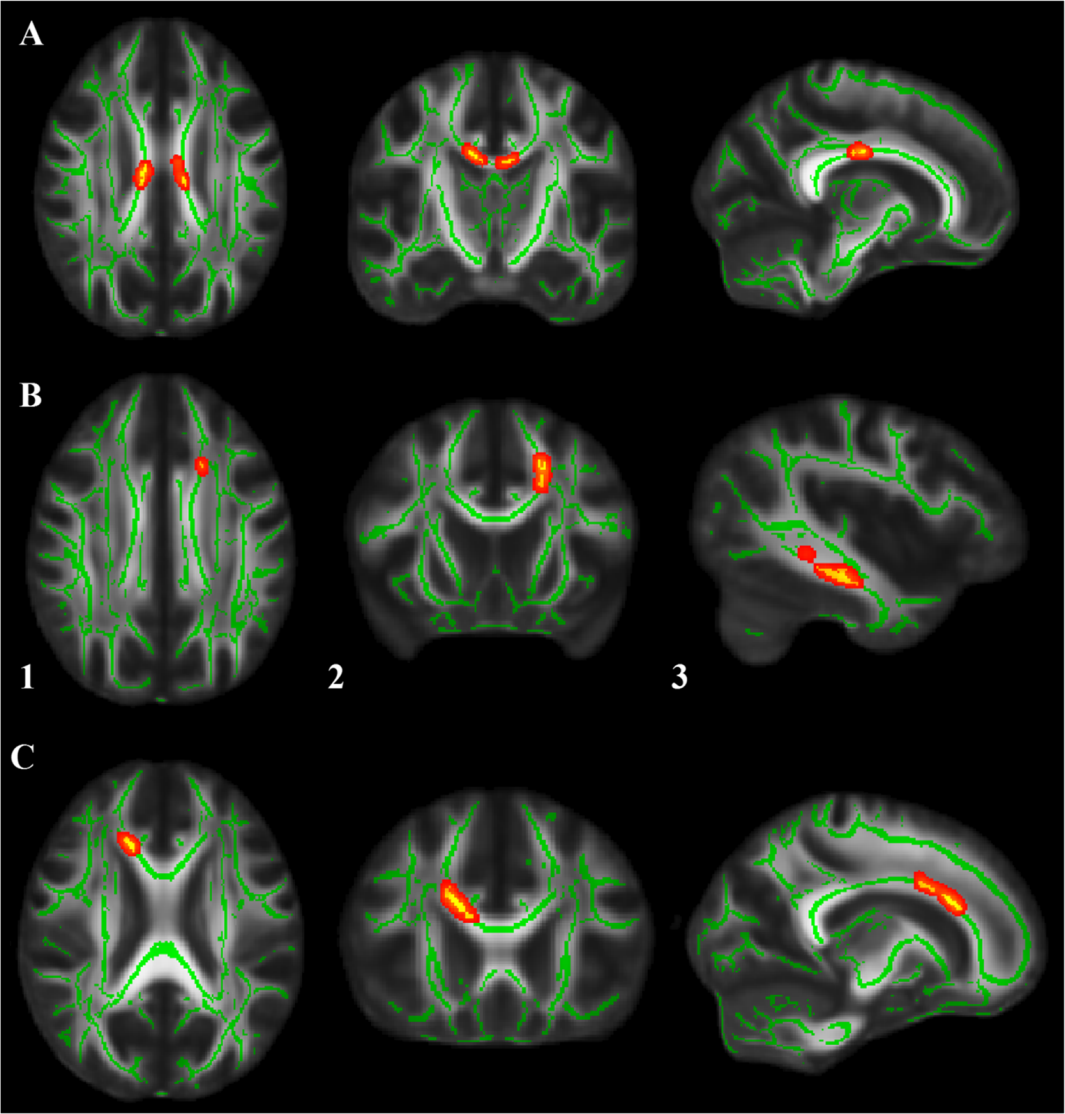
Whole-brain tract-based spatial statistics results, overlaid on axial, coronal and sagittal sections of the white matter skeleton (*green*), showing clusters (*yellow/orange*) of: **a** reduced fractional anisotropy in 47,XXY relative to controls in the body of the corpus callosum; **b** reduced radial diffusivity in 47,XXY relative to controls in (1 + 2) the left anterior corona radiata and (3) the sagittal striatum; **c** reduced axial diffusivity in 47,XXY relative to controls in genu of the corpus callosum. For better visibility, results were thickened using the ‘tbss-fill’ command

As summarized in Table 1 and depicted in Fig. 1, whole-brain TBSS analysis revealed that, compared with controls, the 47,XXY group had significantly lower FA values in the body of the corpus callosum, coupled with significantly lower Da values in the genu of the corpus callosum. In addition, the 47,XXY group had significantly lower Dr. values in the left anterior corona radiata and sagittal striatum. There were no significant differences in MD values between the 47,XXY group and controls.

### TBSS: 47,XXY versus ASD

**Fig. 2:**
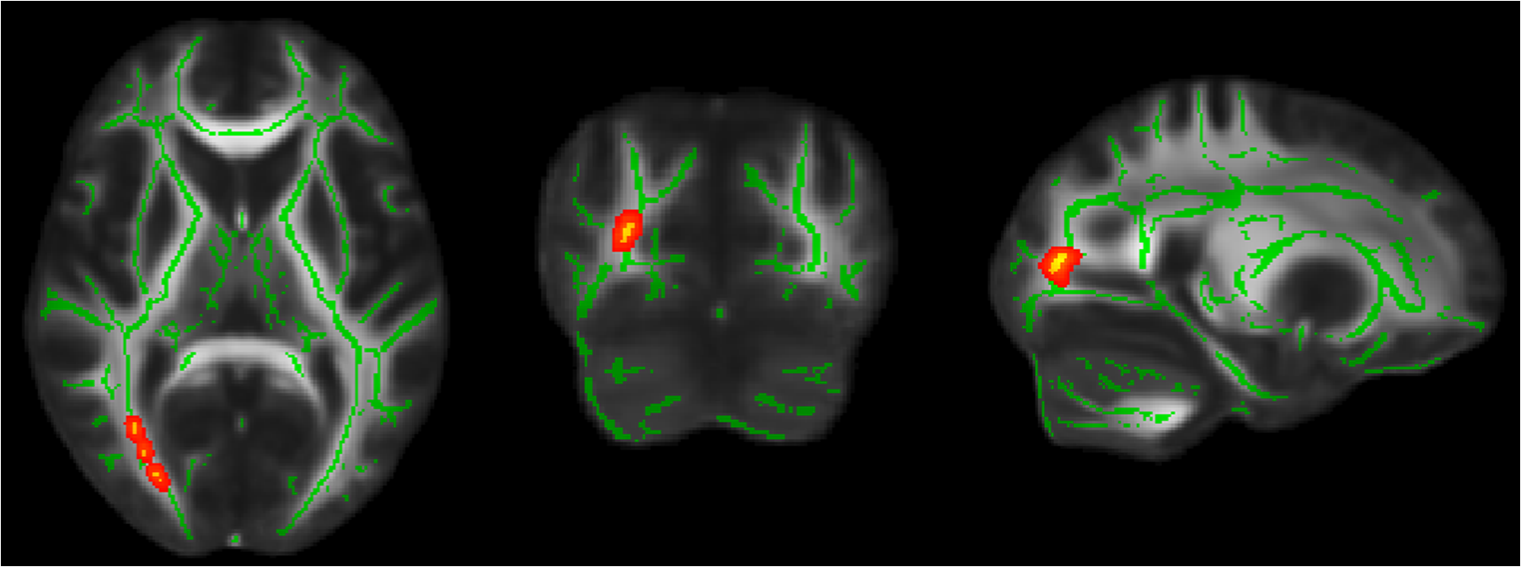
Whole-brain tract-based spatial statistics results, overlaid on axial, coronal and sagittal sections of the white matter skeleton (*green*), showing a cluster (*yellow/orange*) of reduced axial diffusivity in 47,XXY relative to autism spectrum disorders, in the right inferior fronto-occipital fasciculus. For better visibility, results were thickened using the ‘tbss-fill’ command

As summarized in Table 2 and depicted in Fig. 2, whole-brain TBSS analysis revealed that, compared with the ASD group, the 47,XXY group had significantly lower Da values in the right inferior fronto-occipital fasciculus. There were no significant differences in FA, Dr. or MD values between the 47,XXY and ASD groups Table 3.

**Table 2:** Characteristics of clusters of significantly reduced fractional anisotropy, radial diffusivity, and axial diffusivity in 47,XXY relative to controls (TFCE corrected *p* < 0.05)

Measure	#voxels	*p*	Max *t*	x,y,z	Location
Fractional anisotropy	58	0.016	4.56	11,-14,29	Body of corpus callosum
	51	0.019	4.10	−7,-11,27	Body of corpus callosum
Radial diffusivity	3027	0.031	3.79	−18,16,32	Left anterior corona radiata
	730	0.032	4.51	−43,-24,-15	Sagittal striatum
Axial diffusivity	242	0.030	4.84	16,23,24	Genu of corpus callosum

**Table 3:** Characteristics of a cluster of significantly reduced axial diffusivity in 47,XXY relative to autism spectrum disorders (TFCE corrected *p* < 0.05)

Measure	#voxels	*p*	Max *t*	x,y,z	Location
Axial diffusivity	241	0.042	5.23	25,-80,10	Right inferior fronto-occipital fasciculus

## Discussion

This preliminary and exploratory diffusion tensor imaging (DTI) study used tract-based spatial statistics (TBSS) (Smith et al. 2006) to exploratively assess white matter integrity in boys with 47,XXY (also known as Klinefelter syndrome) relative to non-clinical controls, as well as boys with autism spectrum disorders (ASD). The results show that boys with 47,XXY have significantly reduced fractional anisotropy (FA) in the body of the corpus callosum compared with controls, coupled with significantly reduced axial diffusivity (Da) in the genu of the corpus callosum. In addition, boys with 47,XXY show significantly lower radial diffusivity (Dr) in the left anterior corona radiata, and sagittal striatum. No significant differences in mean diffusivity (MD) between boys with 47,XXY and controls were found. In comparison with boys with ASD, boys with 47,XXY show significantly reduced Da in the right inferior fronto-occipital fasciculus. No significant differences in FA, Dr., or MD were found between these groups.

The finding of reduced FA and Da in the corpus callosum in 47,XXY is relevant, as this may help in formulating hypotheses regarding possible mechanisms underlying deficits associated with this condition. In previous studies an association was found between reduced FA in the corpus callosum and reduced performance on bimanual motor coordination (Gooijers and Swinnen 2014). Additionally, a study by Hofer and Frahm (2006) implies the specific callosal regions found to have reduced FA and Da in the current study, i.e. the genu and body, are anatomically connected to cortical (pre- and supplementary) motor regions. Research in individuals with 47,XXY suggests motor dexterity is one of the domains of impairment associated with this condition (Boone et al. 2001). As this is a cognitive domain that is particularly related to the ability for cooperation between hemispheres, the current finding of reduced FA in the corpus callosum is in line with the behavioral phenotype associated with 47,XXY. Results from the current study are supported by findings from a recent study of corpus callosum morphology in males with 47,XXY (Wade et al. 2014), that suggest the presence of morphological changes in the corpus callosum that are related to chromosome dosages. The authors report the most notable effect of chromosome dosages in corpus callosum fiber tracts towards the pre-motor, supplementary motor, and primary motor cortices, the same areas that appear to be affected in our sample.

In addition, Hofer and Frahm (2006) suggest the genu of the corpus callosum is connected to the prefrontal cortex, which includes important language areas, and callosal lesions have been associated with alexithymia and language problems (Devinsky and Laff 2003). Although reduced FA and Da do not equal damaged white matter tracts, it is an indication of reduced integrity of white matter, and thus diminished efficiency of neural connections. Hypothetically, the alexithymia and language problems that have been reported in individuals with 47,XXY (Boada et al. 2009; Leggett et al. 2010; van Rijn et al. 2006) might be in part related to reduced efficiency of interneuronal communication. In support of this hypothesis, in the intact brain the corpus callosum plays an important role in language functions, possibly through facilitation of interhemispheric communication between the left and right plana temporale (Bloom and Hynd 2005; Van der Knaap and Van der Ham 2011). Interestingly, the superior temporal gyri (part of the plana temporale) show decreased functional asymmetry in 47,XXY during language processing (van Rijn et al. 2008). The results from the current study tentatively suggest this functional abnormality may be mediated by deviations in callosal integrity, and are an important starting point for future studies focused on specific tract structures and their association with cognitive and behavioral measures. The only other DTI study in (adult) males with 47,XXY thus far, reported reduced FA in the left posterior limb of the internal capsule, the bilateral anterior cingulate, and the left arcuate bundle (DeLisi et al. 2005). This implies that DTI findings in studies with adults and children differ. To what degree this is related to developmental factors, or to more technical factors (different imaging analysis methodologies were employed), remains unclear. More research in larger samples is necessary to more specifically assess the overlap and differences in white matter microstructure between children and adults with 47,XXY.

The reductions in Dr. seem counterintuitive at first, as clinical conditions are often accompanied by reduced myelin integrity expressed by increased Dr. There may be many genetic or hormonal mechanisms underlying reduced Dr. in individuals with XXY, and we can merely speculate. Nonetheless, there are some relevant findings that may stimulate further research in this area. It has been found that steroid hormones enhance myelination in the human brain (Peper et al. 2011). Hypothetically, testosterone treatment in individuals with 47,XXY could influence myelination. Unfortunately, in the current study the 47,XXY group was too small to assess differences between boys who did receive treatment, and those who did not. However, the finding of reduced Dr. in this group warrants further research focused on the potential neural benefits of testosterone treatment in 47,XXY. This suggestion for future research is strengthened by the finding that testosterone supplements may stimulate the Akt signaling pathway (White et al. 2013), a neural pathway that promotes growth and survival in cells. Flores et al. (2008) point out that enhanced stimulation of the Akt signaling pathway may lead to enhanced myelination. Interestingly, research by Schulz et al. (2014) suggests that the presence of two X chromosomes may stimulate the Akt signaling pathway, which calls for further research to assess if overexpression of such genes may play a role in increased myelination in 47,XXY as well At this time these hypotheses are strictly speculative, as studies investigating these genetic mechanisms in sex chromosome aneuploidies are currently lacking. The results from the current study might inspire future studies in this domain.

As this is the first report of altered white matter microstructure in children with 47,XXY, there is no previous literature that may help understand this counterintuitive finding of lower Dr. in this specific sample. Interestingly, reduced Dr. was also found by Cheng et al. (2010) in adolescents with ASD. The authors imply that individuals with autism may go through a phase of accelerated brain growth early in life, followed by a phase of decelerated brain growth. It would therefore be interesting to incorporate a more developmental view on brain architecture, specifically structural connectivity, in larger samples of individuals with 47,XXY, rather than studying DTI parameters averaged across an age range.

A cluster of significantly reduced Da was found in the right inferior fronto-occipital fasciculus in boys with 47,XXY, compared with boys with ASD. The function of this structure is subject to debate, but it has been implicated in a multitude of domains (e.g. sensory-motor integration, as well as semantic and emotional processing) (Sarubbo et al. 2013). Although both boys with 47,XXY and boys with ASD may experience social difficulties, results from the current study add to previous research suggesting there may be differences in the cognitive and neural characteristics between these groups (Brandenburg-Goddard et al. 2014; Bruining et al. 2010; Van Rijn et al. 2014a; Van Rijn et al. 2014b), one of which being white matter microstructure. These differences underline the need for studies focused on the question of how such underlying (neural) mechanisms relate to social dysfunction in different populations, e.g. children with ASD in the context of 47,XXY, versus children with iodiopathic ASD. This will not only aid in specifying the type of deficit, and in creating awareness that social dysfunction may arise as a consequence of various types of dysfunctions, but it may also have clinical implications. Children with different pathways to social dysfunction might benefit from tailored treatment, which may be developed based on findings from these types of studies. Further research in this area is necessary, preferably focused on individuals with idiopathic ASD versus individuals with an ASD diagnosis in the context of 47,XXY, as the behavioral phenotype in 47,XXY is variable and only a subgroup will meet clinical criteria for ASD. These types of studies may aid in establishing specific (endo)phenotypes that could serve as starting points for intervention studies.

A limitation of the current study was that the ASD group was relatively high functioning in comparison to intellectual functioning typically found in the ASD spectrum (Chakrabarti and Fombonne 2005) due to the demands associated with participating in imaging research, such as having to lie very still in a confined space. Additionally, sample size was small, especially of the 47,XXY group, because of which this study should be considered preliminary and exploratory. However, all results from DTI analysis were corrected for multiple comparisons, meaning significant results were powerful enough to show up even in these small groups. Because this was the first study assessing structural connectivity differences between these populations, no correction was applied for the fact that four measures of white matter integrity were used. However, this study has garnered important results that may give clear direction to future studies in this area.

Taken together, although preliminary, the results from the current study suggest 47,XXY may be associated with significant changes in white matter microstructure. Additionally, the finding of reduced axial diffusivity compared with boys with ASD adds to existing literature suggesting that even though individuals with 47,XXY and those with ASD may share a number of defining characteristics, albeit to varying degrees, distinct differences are also present. This calls for further studies investigating individual variability in underlying neural mechanisms, and therefore different routes to social dysfunction, in 47,XXY versus ASD. Such studies may aid in disentangling these complex gene-brain-behavior relationships.
